# ATP6AP1 as a potential prognostic biomarker in CRC by comprehensive analysis and verification

**DOI:** 10.1038/s41598-024-54437-7

**Published:** 2024-02-18

**Authors:** Shijie Zhang, Yan Wang, Xiaodong Zhang, Min Wang, Hao Wu, Yuwen Tao, Wentao Fan, Li Liu, Bangting Wang, Wenqing Gao

**Affiliations:** 1 Digestive Endoscopy Department, The First Affiliated Hospital with Nanjing Medical University and Jiangsu Province Hospital, Nanjing, Jiangsu China; 2 State Key Laboratory of Genetic Engineering, School of Life Sciences, Shanghai Engineering Research Center of Industrial Microorganisms, Fudan University, Shanghai, 200438 China; 3 Department of Cardiology, The Affiliated Hospital of Jiangnan University, No.1000, He Feng Road, Wuxi, 214122 Jiangsu Province China; 4 The Friendship Hospital of Ili Kazakh Autonomous Prefecture, Ili & Jiangsu Joint Institute of Health, Yining, China

**Keywords:** ATP6AP1, CRC, Biomarker, Immune infiltration, Prognosis, Cancer, Cancer genetics, Gastrointestinal cancer, Tumour biomarkers

## Abstract

The role of ATP6AP1 in colorectal cancer (CRC) remains elusive despite its observed upregulation in pan-cancer. Therefore, the current study aimed to assess the clinical significance of ATP6AP1 and its relationship with the immune infiltration in CRC. Transcriptome data of CRC were obtained from The Cancer Genome Atlas (TCGA) database and analyzed using the combination of R packages and tumor-related databases, including TIMER2, TISIDB, cBioPortal, and MethSurv. The tissue arrays and immunohistochemical staining were performed to verify the expression and clinical characteristics of ATP6AP1. The results revealed that ATP6AP1 expression was significantly elevated in CRC and associated with poor clinicopathological characteristics and prognosis. Furthermore, the analysis demonstrated ATP6AP1 expression was correlated with the infiltration of immune cells and cancer-associated fibroblasts in the microenvironment of CRC. Moreover, ATP6AP1 was found to be linked to various immune checkpoints and chemokines, with enrichment of cytoplasmic vesicle lumen, endopeptidase regulator activity, and endopeptidase inhibitor activity observed in the high ATP6AP1 expressional group. In conclusion, the findings of this study suggest that ATP6AP1 upregulation may serve as a biomarker for poor diagnosis in CRC and offer a potential target for immunotherapy in CRC.

## Introduction

Colorectal cancer (CRC) is a heterogeneous disease in terms of cytology and molecular biology, including colon cancer (COAD) and rectal cancer (READ), which ranked third in morbidity and second in tumor-related death worldwide in 2018^1^. The incidence and mortality of CRC gradually rise with age, which brings a significant burden to the public^2^. In particular, the incidence of early-onset colorectal cancer has increased dramatically among patients aged 20 to 49 over the last 25 years^3,4^. By 2030, ten percent of colon and twenty-five percent of rectal cancers will be diagnosed in individuals younger than 50 years^5^. CRC patients typically have microsatellite stability/instability and exhibit long interspersed nuclear elements (LINE-1) hypomethylation and gene sequence variations, including TP53, KRAS, BRAF, and APC^6–8^. Currently, the treatment strategies for CRC mainly include neoadjuvant chemotherapy, surgical resection, and targeted therapy, which have favorable effects on CRC^9–11^. However, the death rate of CRC remains high for postoperative recurrence and metastasis. The occurrence and progression of CRC are accompanied by numerous changes in gene expression and the activation of multiple signaling pathways, which lead to different clinical and pathological features. Various therapeutic agents have been developed based on genetic mutations and pathway activation, resulting in diverse outcomes for CRC patients, such as treatment sensitivity or drug resistance. Nowadays, the molecular expression profile of CRC has been further explored in depth with the rapid development of next-generation sequencing technology, which allows us to utilize these molecular biomarkers as diagnostic criteria, therapeutic targets, and prognostic risk factors for CRC patients.

The ATP6AP1 gene encodes for ATPase H + transporting protein. Abnormal ATP6AP1 expression may promote congenital diseases and carcinogenesis. ATP6AP1 gene mutations are associated with congenital disorders of glycosylation (CDG) and can affect multiple organ systems^12^. Descriptions of postnatal phenotype include immunodeficiency, hepatopathy, and cognitive impairment^13,14^. ATP6AP1 was upregulated in breast cancer tissues, and higher ATP6AP1 expression was associated with poorer outcomes. Besides, ATP6AP1 levels exhibited significant negative correlations with B cells, CD8 + T cells, macrophages, and Treg cells (p < 0.05)^15^. Based on immune gene expression in ER (+) and/or PR (+) and HER2 (−) BC, Peng Yuan et al. constructed a 7-gene prognostic signature that displayed distinct patterns of prognosis and genomic features, and ATP6AP1 was one of the seven signature genes^16^.

This study aimed to analyze the ATP6AP1 expression profile and explore its potential prognostic value, varied molecular biological functions, and involved signal pathways in CRC. Our study revealed the potential role of ATP6AP1 played in regulating tumor immunity and activating tumor-associated pathways based on bioinformatic analysis. We hope our findings could provide new insights for disease diagnosis and prognosis evaluation, antitumor therapy, and sensitizing immunotherapy for colorectal cancer patients.

## Results

### High expression of ATP6AP1 in colorectal cancer samples

**Figure 1:**
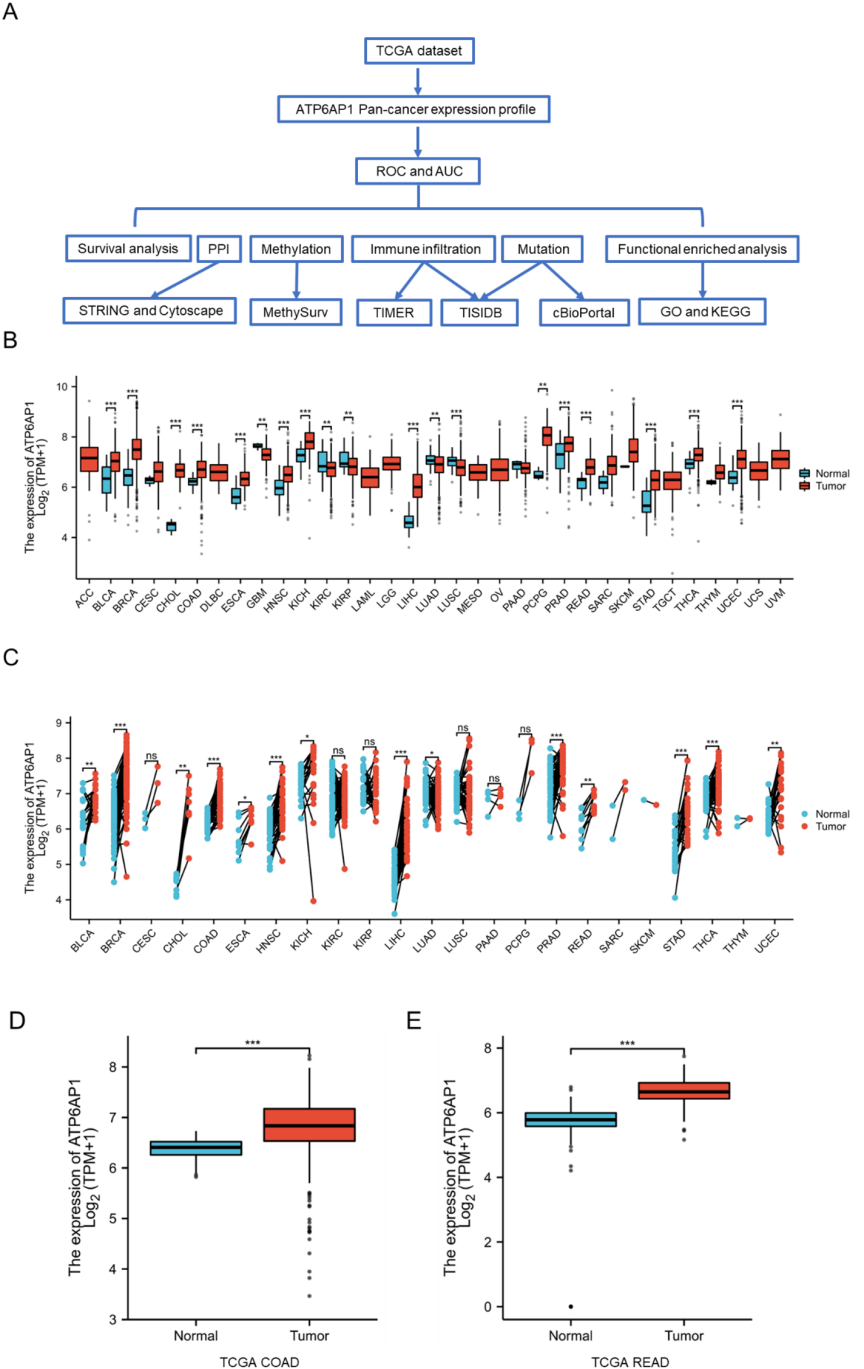
Identify ATP6AP1 as a novel biomarker in colorectal cancer. (**A**) Flow chart of the present research. (**B**) ATP6AP1 pan-cancer expression in different tumor based on the TCGA database. (**C**) Paired ATP6AP1 pan-cancer expression in different tumor based on the TCGA database. (**D**, **E**) ATP6AP1 expression was higher in COAD and READ than in normal tissues based on the TCGA database. ***Indicates p-value < 0.001. *READ* rectal adenocarcinoma, *COAD* colon adenocarcinoma.

To explore the biological function of ATP6AP1 in pan-cancer, especially CRC, we performed a series of bioinformatics analyses (Fig. 1A). First, we analyzed the transcription levels of ATP6AP1 across various cancers by comparing tumor and normal samples using data from the TCGA database (Table S2). In TCGA data, differential ATP6AP1 expression was significant among 19 of the 33 cancer types analyzed, including 14 upregulation and 5 downregulation (Fig. 1B). However, when performing the analysis of matched ATP6AP1 mRNA expression using TCGA data, we found significant differences in 14 out of the 23 cancer types examined (as shown in Table S1) (Fig. 1C). Moreover, ATP6AP1 expression was higher in tumors than in normal tissues in COAD and READ. Based on the TCGA database and UALCAN, we found ATP6AP1 was highly expressed in COAD and READ (Fig. 1D,E and Fig. S1).

### Mutation of ATP6AP1 in COAD and READ

Gene mutations are of great significance to the tumorigenesis and development of cancer. Therefore, we explored the mutations of ATP6AP1 in CRC. The gene alteration rate of ATP6AP1 appears for CRC patients with amplification (> 0.5%) using the cBioPortal database (Fig. S2A). Amplification and miss mutation are the primary type of frequent genetic alterations of ATP6AP1. The types, sites, and case numbers of the ATP6AP1 gene modification were further displayed (CPTAC-2 Prospective, Cell 2019, and TCGA Firehose Legacy) (Fig. S2B,C).

### Association of ATP6AP11 with clinical stage and prognosis

**Figure 2:**
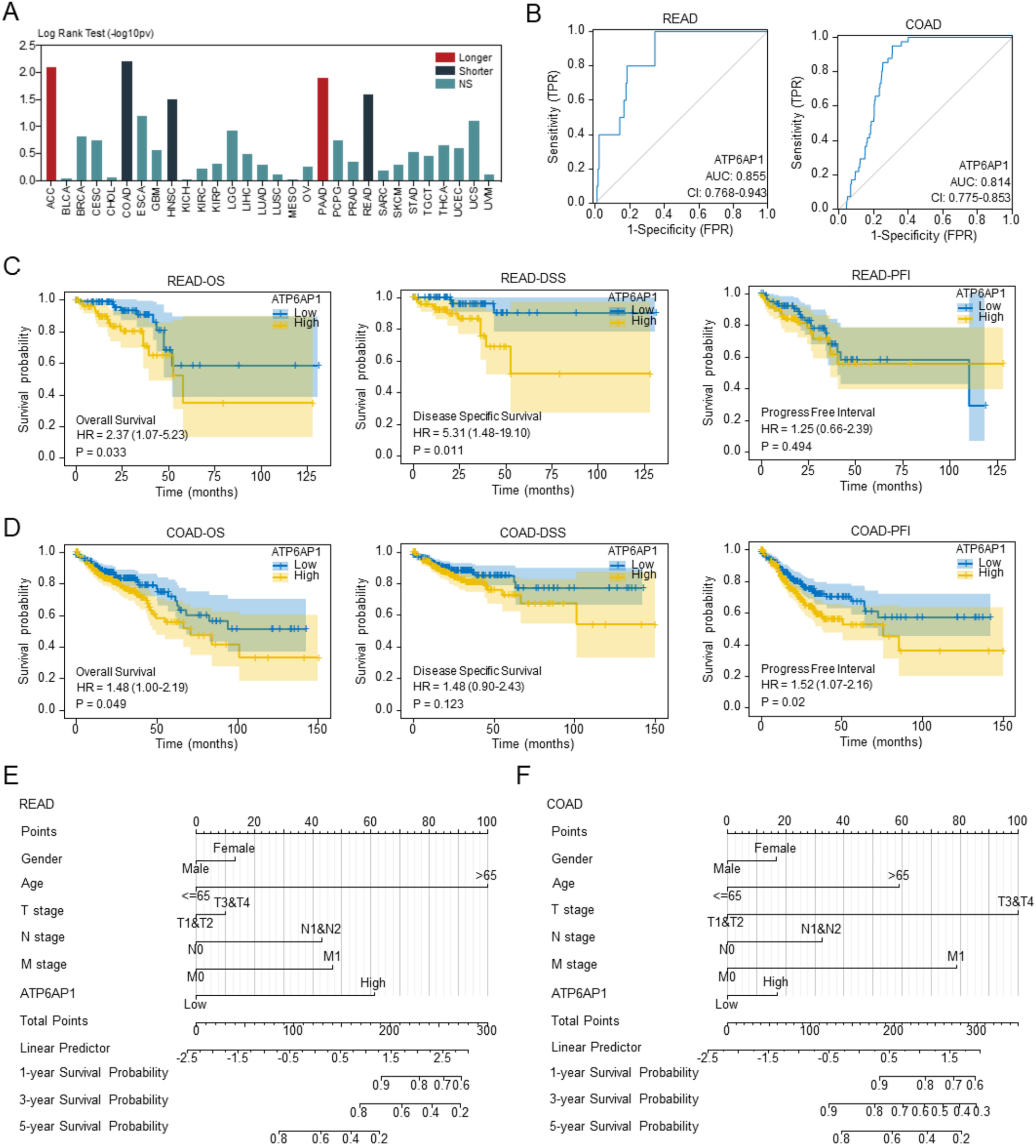
High-expressional ATP6AP1 affects the prognosis of CRC patients. (**A**) Association between ATP6AP1 expression and overall survival across human cancers TISIDB database. (**B**) The ROC and AUC of READ and COAD are based on the expression of ATP6AP1. (**C**) The OS, DSS, and PFI of READ patients between low and high-expressional groups (n = 83). (**D**) The OS, DSS, and PFI of COAD patients between low (n = 238) and high-expressional (n = 239) groups. (**E**, **F**) Nomogram to predict the READ and COAD survival possibility among 1 year, 3 years and 5 years, including UBTD1 and independent clinical risk factors. *READ* rectal adenocarcinoma, *COAD* colon adenocarcinoma, *OS* overall survival, *DSS* disease-special survival, *PFI* progress-free interval.

We analyzed the correlation between ATP6AP1 and clinical stage according to the grouping of high and low ATP6AP1 expression in READ and COAD. In READ cases, ATP6AP1 was not associated with lymph node invasion, distant metastasis, or tumor stage, with the exception of its correlation with patient age (p-value = 0.035). However, in COAD, ATP6AP1 was only positively correlated with distant metastasis (*p*-value = 0.003) (Table 1). Firstly, the overall survival of ATP6AP1 in 30 tumors was constructed via the TISIDB database, among which the OS of ATP6AP1 in READ and COAD was significantly shorter (Fig. 2A). Furthermore, we constructed a diagnostic ROC model based on the expression data of ATP6AP1. We found that the ROC model had a good performance in classifying READ and COAD patients from normal tissues (area under the ROC curve = 0.855, Fig. 2B) and in the validation set (area under the ROC curve = 0.814, Fig. 2B). Next, the Kaplan–Meier (KM) Plotter was utilized to analyze the correlation between the mRNA level of ATP6AP1 and the survival of patients with READ and COAD. The results showed that the increased mRNA level of ATP6AP1 was significantly associated with shorter OS (p < 0.033) and DSS (p = 0.011) in the patients with READ (Fig. 2C). However, the analysis of survival in COAD revealed that high expression of ATP6AP1 was associated with shorter OS (p = 0.049) and PFI (p = 0.012) (Fig. 2D). In addition, we performed a nomogram to predict the survival rates for READ and COAD patients based on age, gender, TNM stage, tumor stage, and risk score. The calibration curves of the nomogram indicated good consistency between the predicted survival rate and actual 1-, 3- and 5-year survival rate (Fig. 2E,F and Fig. S3).

**Table 1:** Clinicopathological characteristics of the patient cohorts.

Characteristic	READ (n = 29)			COAD (n = 29)		
	Low expression of ATP6AP1	High expression of ATP6AP1	P-value	Low expression of ATP6AP1	High expression of ATP6AP1 (n = 239)	P-value
	(n = 83)	(n = 83)		(n = 239)		
T stage, n (%)			0.093			0.941
T1	6 (3.7%)	3 (1.8%)		5 (1%)	6 (1.3%)	
T2	9 (5.5%)	19 (11.6%)		42 (8.8%)	41 (8.6%)	
T3	61 (37.2%)	52 (31.7%)		163 (34.2%)	160 (33.5%)	
T4	5 (3%)	9 (5.5%)		28 (5.9%)	32 (6.7%)	
N stage, n (%)			0.305			0.05
N0	39 (24.1%)	45 (27.8%)		155 (32.4%)	129 (27%)	
N1	20 (12.3%)	25 (15.4%)		48 (10%)	60 (12.6%)	
N2	20 (12.3%)	13 (8%)		36 (7.5%)	50 (10.5%)	
M stage, n (%)			0.945			0.003
M0	59 (39.6%)	67 (45%)		184 (44.3%)	165 (39.8%)	
M1	10 (6.7%)	13 (8.7%)		21 (5.1%)	45 (10.8%)	
Age, median (IQR)	64 (56, 71.5)	66 (59, 74.5)	0.035	69 (60, 78)	68 (57, 77)	0.325

To further confirm the ATP6AP1 prognostic role in COAD and READ patients, we performed univariate and multivariate survival analyses (Tables S1–S2). Univariate analysis identified four prognostic factors: ages (≥ 60 vs. < 60), N (N1–2 vs. N0), M (M1–2 vs. M0), and Stages (III–IV vs. I–II) in READ. Similarly, ages (≥ 60 vs. < 60), BMI (< 25 vs. ≥ 25), N (N1–2 vs. N0), M (M1–2 vs. M0), Stage (III–IV vs. I–II), and CEA level (≥ 5 vs. < 5 mg/ml) were influential univariate prognostic factors for COAD patients. Multivariate survival analysis further uncovered that age (p-value < 0.001) in READ and N stage (p-value = 0.015) in COAD were separately prognostic of OS (Tables S1–S2).

### Correlation analysis of ATP6AP1 and immune-infiltrating cells

**Figure 3:**
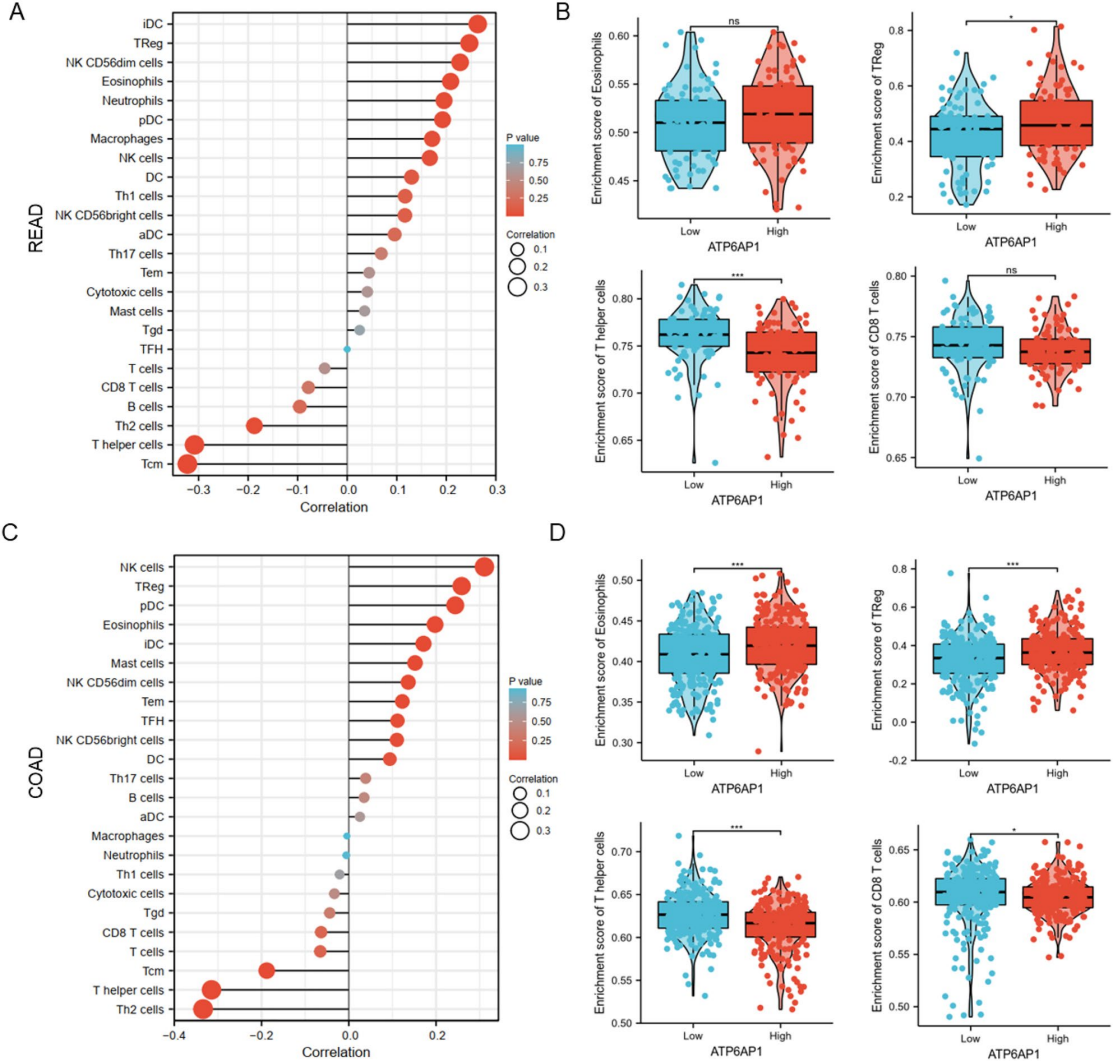
Correlation of immune cell infiltration and ATP6AP1 expression in CRC patients. (**A**) Relationships among infiltration levels of 24 immune cell types and ATP6AP1 expression profiles by Spearman’s analysis in READ. (**B**) The infiltration levels of Eosinophils, Treg cells, T helper cells, and CD8 + cells in the high- and low-ATP6AP1 expression groups in READ. (**C**) Relationships among infiltration levels of 24 immune cell types and ATP6AP1 expression profiles by Spearman’s analysis in COAD. (**D**) The infiltration levels of Eosinophils, Treg cells, T helper cells, and CD8 + cells in the high- and low-ATP6AP1 expression groups in COAD. *DCs* dendritic cells, *aDCs* activated DCs, *iDCs* immature DCs, *pDCs* plasmacytoid DCs, *Th* T helper cells, *Th1* type 1 Th cells, *Th2* type 2 Th cells, *Th17* type 17 Th cells, *Treg* regulatory T cells, *Tgd* T gamma delta, *Tcm* T central memory, *Tem* T effector memory, *Tfh* T follicular helper, *NK* natural killer.

The tumor microenvironment plays a crucial role in tumor genesis and development. Therefore, it is significant to investigate the relationship between ATP6AP1 and immune cell infiltration. Infiltration of 24 immune cell types in COAD and READ was determined by the ssGSEA, and then the relationship between ATP6AP1 and immune cell infiltration was analyzed by Pearman (Tables S4–S5). In READ, iDC (R = 0.27, p < 0.001), Eosinophils (R = 0.224, p = 0.004), and TReg (R = 0.328, p < 0.001) were all positively correlated with ATP6AP1 expression. However, Tcm (R =  − 0.316, p < 0.001) and Th2 cells (R =  − 0.236, p = 0.002) showed a negative association with ATP6AP1 (Fig. 3A). The tumor infiltration levels of T helper cells and Treg cells (Fig. 3B) were consistent with Pearman’s analysis results in Fig. 3A. However, there was no significant difference between high and low ATP6AP1 in Eosinophils and CD8 + T cells. Similarly, in COAD, NK cells (R = 0.512, p < 0.001), iDC (R = 0.317, p < 0.001), and Treg (R = 0.284, p = 0.002) were all positively correlated with ATP6AP1 expression. However, Tcm (R =  − 0.436, p < 0.001), T helper cells (R =  − 0.374, p < 0.001), and Th2 cells (R =  − 0.293, p < 0.001) showed a negative association with ATP6AP1 (Fig. 3C). The tumor infiltration levels of Eosinophils, Treg cells, T helper cells, and CD8 + cells (Fig. 3D) were consistent with Pearman’s analysis results in Fig. 3C.

**Figure 4:**
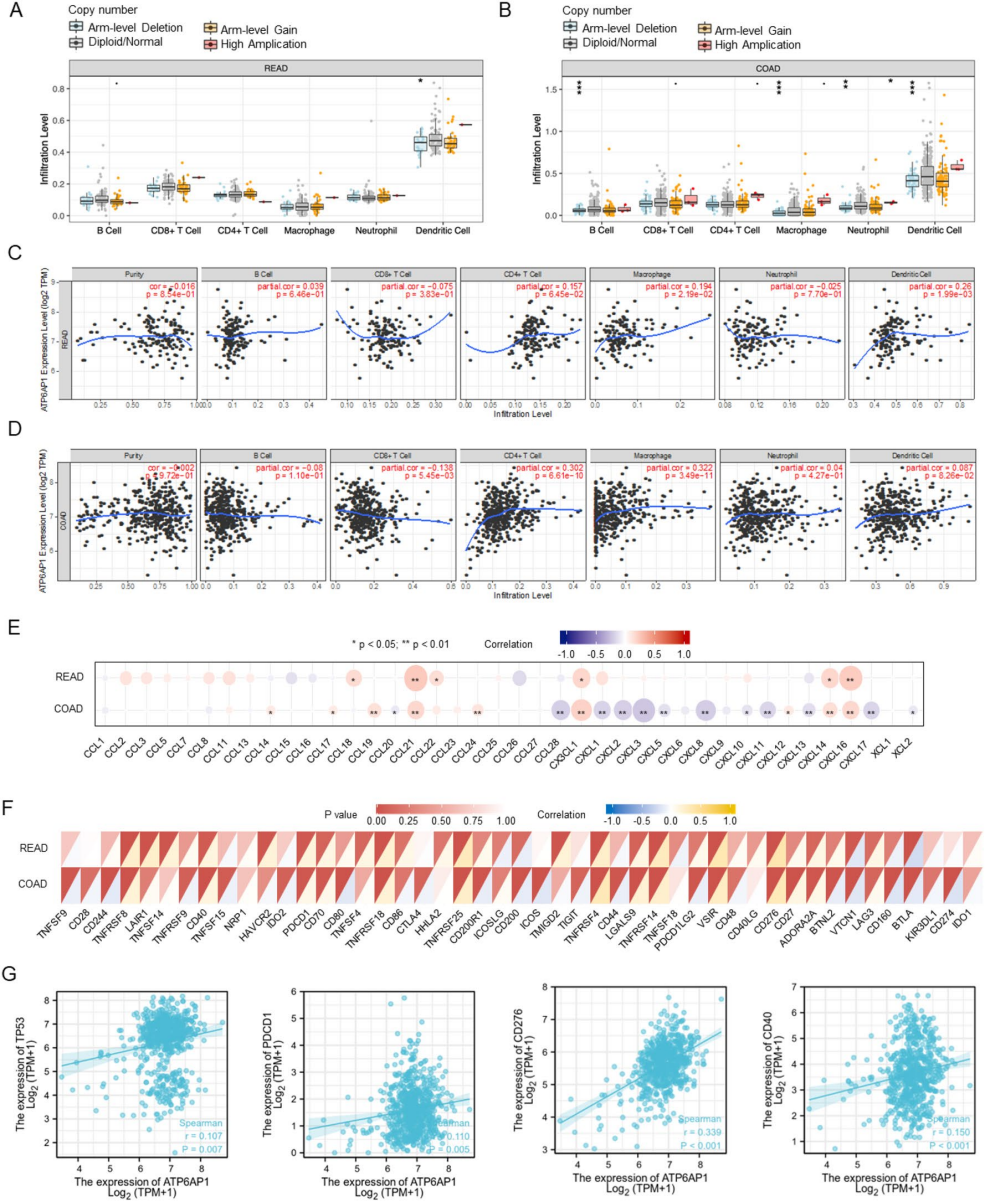
Association between ATP6AP1 and infiltrated inflammatory cells and immune checkpoints was analyzed by the TISIDB database. (**A**, **B**) Correlation analysis between ATP6AP1 gene copy and immune cell (B cell, CD8 + T cell, CD4 + T cell, Macrophage, Neutrophil, and Dendritic cell) infiltration levels in COAD and READ. (**C**, **D**) ATP6AP1 expression is related to immune infiltration levels in COAD and READ. (**E**) Correlations between ATP6AP1 and different chemokines in COAD and READ. READ, rectal adenocarcinoma; COAD, colon adenocarcinoma. (**F**) Correlation analysis of ATP6AP1 expression levels with 40 common immune checkpoint genes in COAD and READ. (**G**) The correlation analysis results between the expression levels of ATP6AP1 and TP53, PDCD-1, CD276, and CD40 in the TCGA-CRC dataset.

Furthermore, we analyzed the relationship between different somatic CNA (copy number aberrations) and tumor immune infiltration using TIMER. Firstly, the “SCNA” module analysis showed that several immune cell infiltration levels seemed to associate with altered ATP6AP1 gene copy numbers in COAD, including B cells, macrophages, neutrophils, and dendritic cells (Fig. 4B). In comparison, ATP6AP1 gene copy numbers were only associated with dendritic cells’ infiltration levels in READ (Fig. 4A). Secondly, the “Gene” module analysis confirmed that the immune infiltration levels of B cells, CD8 + T cells, CD4 + T cells, macrophage cells, neutrophil cells, and dendritic cells were associated with ATP6AP1 expression in READ and COAD (Fig. 4C,D). In conclusion, the high expression of ATP6AP1 is associated with changes in the infiltration proportions of immune cells, potentially influencing the immune microenvironments in both READ and COAD.

Chemokine networks affect tumor immunity and tumorigenesis by regulating the tumor microenvironment. Therefore, we next performed the correlations between chemokines and ATP6AP1 in CRC. We analyzed the correlations between ATP6AP1 expression and immune checkpoint molecules using spearman’s method. The results showed significantly (p < 0.05) and strong positive correlations in the following chemokines: CCL21, CXCL16, CXCL14, CX3CL1, CCL18, and CCL22 (Fig. 4E). Besides, strong negative correlations with chemokines, including CXCL1-2/8, CXCL11, CXCL17, CXCL13, and CXCL5 in COAD. Therefore, these results further confirmed that the ATP6AP1 was significantly correlated with immune infiltrating cells in READ and COAD, indicating that ATP6AP1 may play a crucial role in the READ and COAD microenvironment.

### Relationship between ATP6AP1 and immunosuppressive molecules

The results showed that ATP6AP1 expression levels were significantly related to most immunosuppressive molecules, including TNFRSF8, CD40, PDCD1, TNFRSF18, TNFRSF25, TNFRSF4, TNFRSF14, VSIR, CD276, VTCN1, and BTLA in CRC (all p < 0.05, Fig. 4F). Furthermore, we analyzed correlations between ATP6AP1 and different chemokines in COAD and READ. ATP6AP1 expression was a positive relationship with TP53, PDCD1, CD276, and CD20 in CRC (Fig. 4G). These results suggested that ATP6AP1 might cause immunosuppression in the CRC microenvironment.

### Correlation analysis of ATP6AP1 and cancer-associated fibroblasts

**Figure 5:**
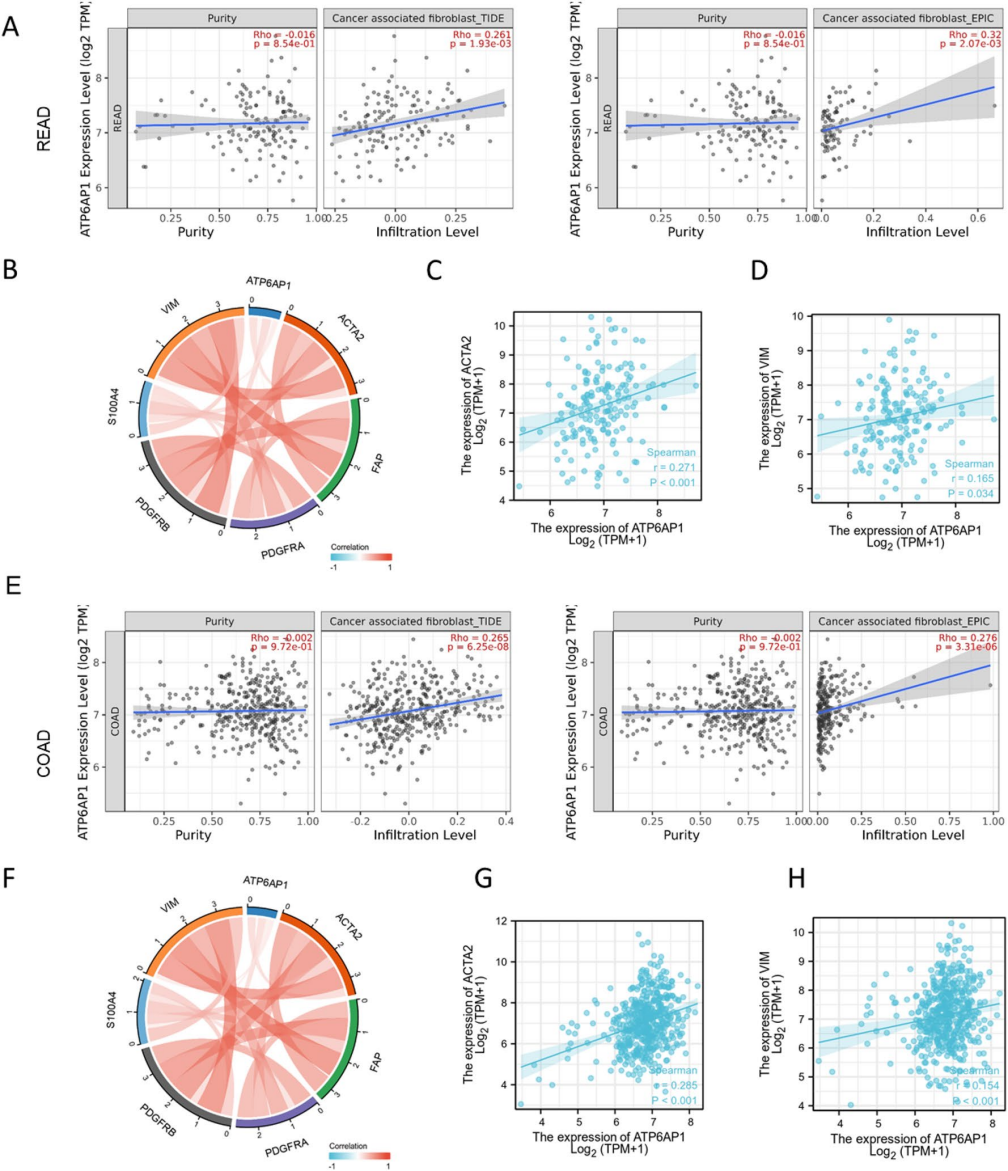
Correlation analysis of ATP6AP1 and Cancer-associated fibroblasts. (**A**) The association of ATP6AP1 and cancer-associated fibroblast in READ by TIDE (p-value = 1.93e−03) and EPIC (p-value = 2.07e−03). (**B**–**D**) The correlation analysis between the ATP6AP1 expression and the expression levels of ACAT2, S1004A, VIM, and PDGFRA/B in the TCGA-READ dataset. (**E**) The association of ATP6AP1 and cancer-associated fibroblast in COAD by TIDE (p-value = 6.25e−08) and EPIC (p-value = 3.31e−06). (**F**–**H**) The correlation analysis between the ATP6AP1 expression and the expression levels of ACAT2, S1004A, VIM, and PDGFRA/B in the TCGA-COAD dataset.

Cancer-associated fibroblasts (CAFs) constitute a favorable environment for tumor development. CAFs can inhibit immune cell function by secreting various cytokines or metabolites and promoting tumorigenesis, invasion, metastasis, and drug resistance. We analyzed the correlation between ATP6AP1 and CAFs through TIMER and found that there was a significant positive correlation between them in COAD (p = 1.93e−03 in TIDE and p = 2.07e−03 in EPIC) and READ (p = 6.25e−08 in TIDE and p = 3.31e−06 in EPIC) (Fig. 5A,E). High ATP6AP1 expression was positively related to the markers of CAFs, such as ACAT2, S1004A, VIM, and PDGFRA/B in COAD and READ (Fig. 5B,F). In COAD, ATP6AP1 expression was positive relation with ACAT2 (p-value < 0.001) and VIM (p-value = 0.004) (Fig. 5G,H). Similarly, ATP6AP1 expression was positive relation with ACAT2 (p-value < 0.001) and VIM (p-value < 0.001) in READ (Fig. 5C,D).

### The methylation status of the ATP6AP1 is associated with the prognosis of COAD and READ patients

DNA methylation levels in the ATP6AP1 and the prognostic value of the CpG islands in the ATP6AP1 were analyzed using the MetSurv tool. The results showed 10 methylated CpG islands in COAD, including cg02893780 and cg24332600, which exhibited elevated DNA methylation levels (Fig. S4). We also observed 11 methylated CpG islands in READ, including cg02893780, cg10645377, and cg24332600, showing high DNA methylation levels (Fig. S5). Furthermore, methylation levels of four CpG islands, named cg00645049, cg17800714, cg18742441, and cg19168249, were associated with the prognosis (p < 0.05) in COAD (Table S4). However, no CpG islands in READ related to the prognosis (Table S5). Elevated levels of ATP6AP1 methylation in these four CpG islands were associated with poorer overall survival of COAD patients compared to those with lower levels of CpG methylation.

### Screening DEGs and functional enrichment analyses based on ATP6AP1 single gene analysis

**Figure 6:**
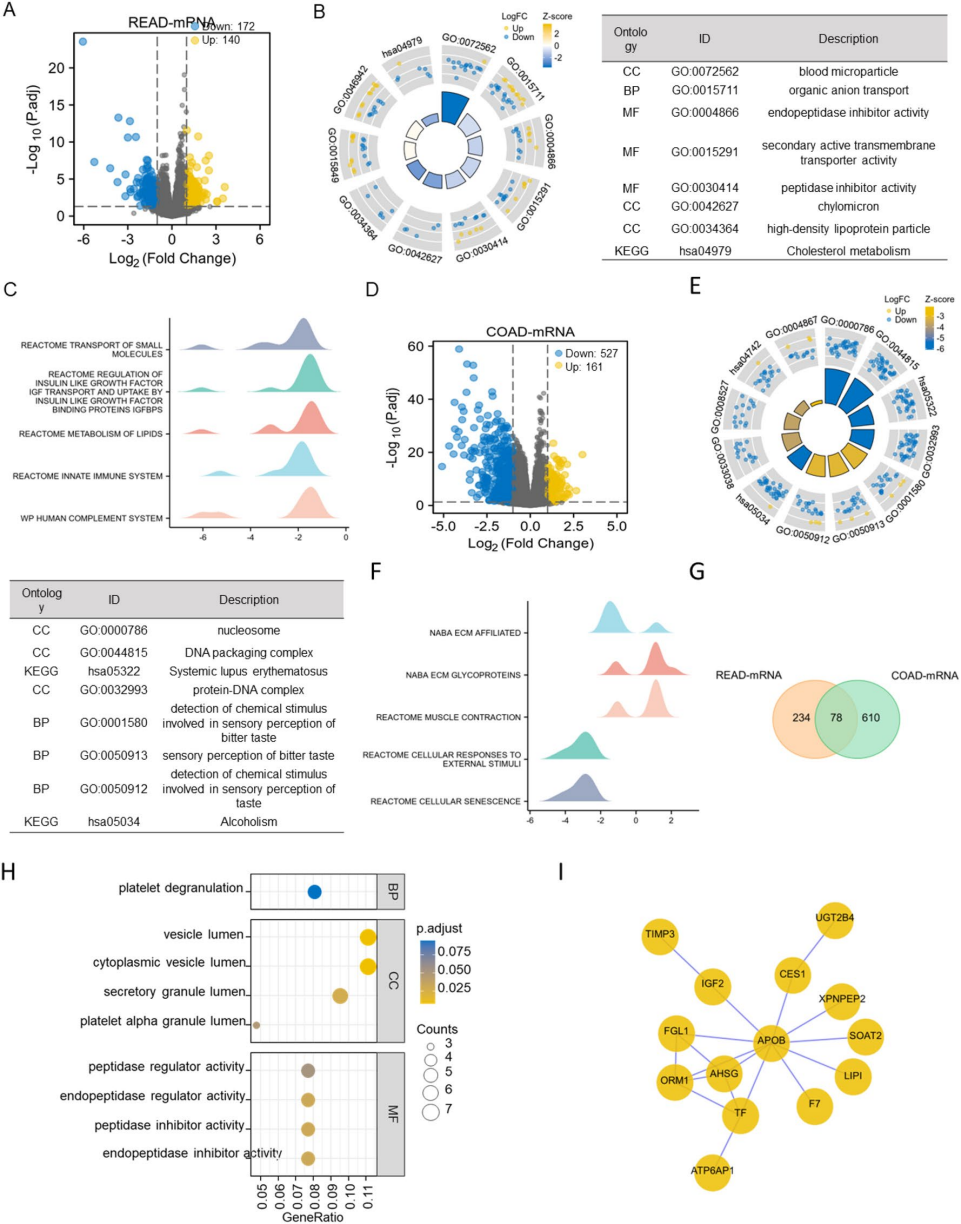
Screening DEGs and constructing functional analysis between ATP6AP1 high-expressional group and ATP6AP1 low-expressional group. (**A**) Volcano plots of DEGs between the expression of ATP6AP1-high and ATP6AP1-low in READ samples. (**B**) GO and KEGG analysis among DEGs in READ as a circle graph. (**C**) GSEA analysis of DEGs between ATP6AP1-high and ATP6AP1-low in READ samples. (**D**) Volcano plots of DEGs between the expression of ATP6AP1-high and ATP6AP1-low in COAD samples. (**E**) GO and KEGG analysis among DEGs in COAD as a circle graph. (**F**) GSEA analysis of DEGs between ATP6AP1-high and ATP6AP1-low in COAD samples. (**H**) Volcano plots of common DEGs between COAD and READ samples based on (**A**) and (**C**). (**I**) PPI network of common DEGs performed by STRING. (**J**) GO and KEGG functional analysis of common DEGs. *DEGs* differential expressional genes, *READ* rectal adenocarcinoma, *COAD* colon adenocarcinoma.

Based on the TCGA database, we conducted ATP6AP1 single gene analysis, divided the tumor samples into ATP6AP1 low expression group and ATP6AP1 high expression group by R (version 4.1.1), and screened differentially expressed genes. The 30 most significant DEGs between the two cohorts are shown in Fig. S6. 668 DEGs, including 359 upregulated and 319 downregulated genes, were identified between low and high ATP6AP1 groups in READ (Fig. 6A). 6943 DEGs, including 228 upregulated and 6715 downregulated genes, were identified between low and high ATP6AP1 groups in COAD (Fig. 6D). The potential bio-function of DEGs was explored via GO and KEGG enrichment analysis. Organic anion transport, high-density lipoprotein particle, and cholesterol metabolism were enriched in READ via GO and KEGG analysis (Fig. 6B). Besides, GO analysis revealed that nucleosome, DNA packaging complex, and protein-DNA complex were enriched in COAD (Fig. 6E). ATP6AP1 may promote CRC by increasing cholesterol metabolism and DNA synthesis. GSEA showed that the ATP6AP1-associated DEGs were significantly enriched in regulating insulin-like growth factors, innate immune system, and metabolism of lipids in READ (Fig. 6C). ATP6AP1-associated DEGs significantly correlated with the NABA ECM affiliated, NABA ECM glycoprotein, and cellular senescence in COAD (Fig. 6F).

Using Venn diagrams, we screened out 78 common differential expressional genes in READ and COAD (Fig. 6G). Next, we constructed PPI networks of the 78 DEGs using the STRING protein database and screened cluster 1 module, including 14 nodes and 18 edges (Fig. 6H). To explore the molecular mechanisms underlined, GO and KEGG pathway enrichment analyses of common DEGs were performed (Fig. 6I). GO analysis, including biological processes, cellular components, and molecular functions, were displayed in the bar plot. Cytoplasmic vesicle lumen, peptidase inhibitor activity, peptidase inhibitor activity, and endopeptidase regulator activity were enriched in DEGs.

### High ATP6AP1 expression is strongly associated with CRC prognosis

**Figure 7:**
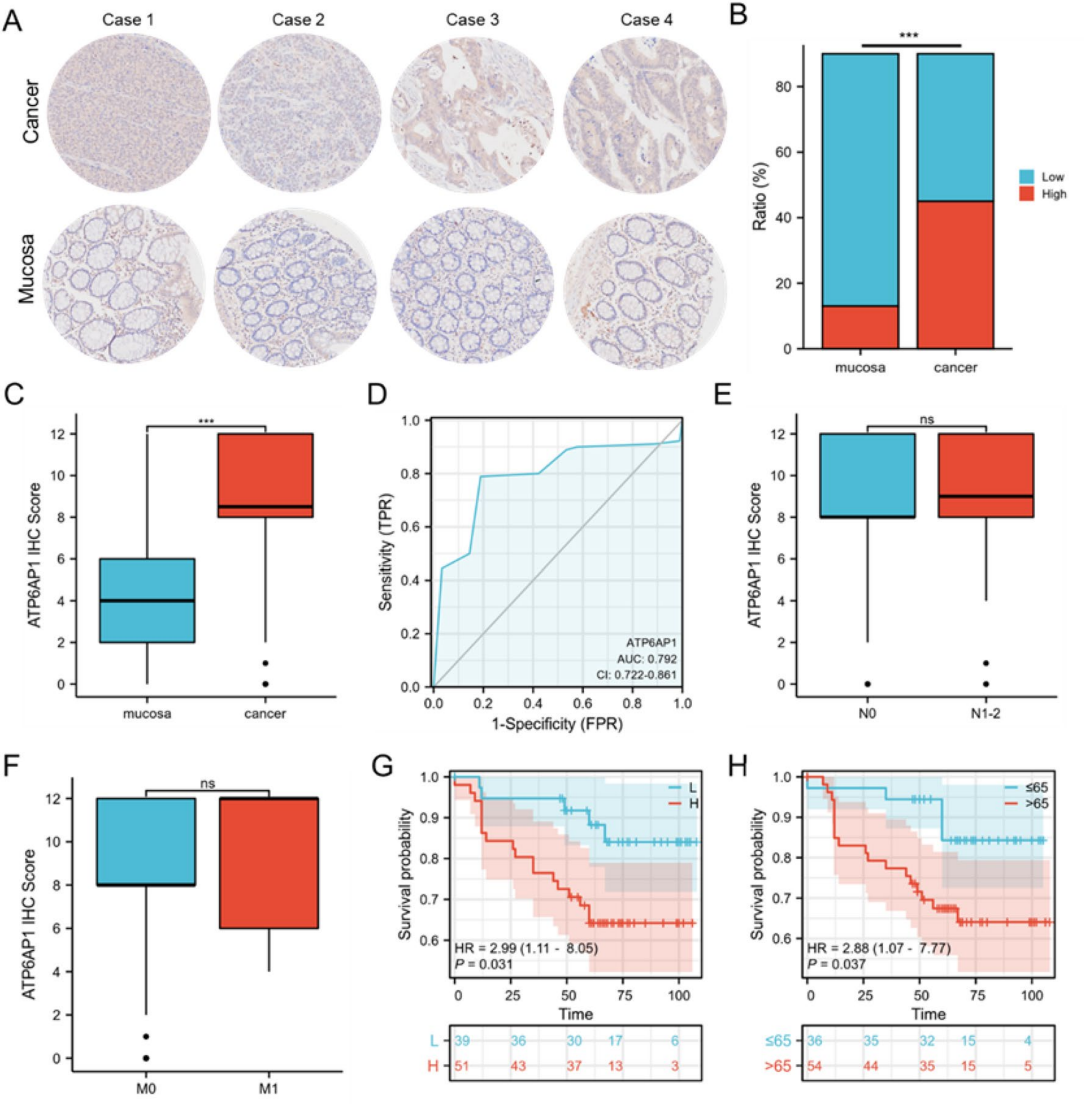
High expression of ATP6AP1 predicts poor prognosis in CRC patients. (**A**) IHC analysis of ATP6AP1 expression in a CRC patient tissue array (n = 90). ATP6AP1 levels in cancer tissues and corresponding normal tissues. Representative images are shown (10x). (**B**) The percent of high and low ATP6AP1 expression in CRC patients (normal and cancer tissues). (**C**) IHC scores of ATP6AP1 in human CRC tissues and adjacent tissues. (**D**) ROC curve with corresponding AUC value for ATP6AP1 when classifying cancer from the mucosa. (**E**, **F**) ATP6AP1 expression in 90 CRC patients was analyzed based on the following parameters: lymph node invasion status and tumor metastasis. Blue: adjacent tissues; Red: cancers. (**G**) High IHC scores of ATP6AP1 predict poor OS [p-value = 0.031, HR 2.99 (1.11–8.05)]. L: low-expressional ATP6AP1; H: high- expressional ATP6AP1. (**H**) High age predicted poor OS [p-value = 0.037, HR 2.88 (1.07–7.77)]. Data was shown as mean and standard deviation from three independent experiments. Data are means ± SDs. *p < 0.05; **p < 0.01; ***p < 0.001.

We finally examined the functions of ATP6AP1 in CRC by exploring the relative expression of ATP6AP1 in CRC patient tissues and corresponding adjacent tissues. ATP6AP1 has been expressed differently in 90 patients’ specimens (Fig. 7A). According to IHC scores, we classified the specimens into high and low ATP6AP1 expression groups. High ATP6AP1 expression accounted for a higher proportion in tumor tissues (Fig. 7B). The amount of ATP6AP1 was obviously increased in CRC compared with adjacent mucosa (Fig. 7C). Then, we utilized ROC and AUC to predict the diagnostic value of ATP6AP1 in differentiating CRC from the mucosa. The AUC value of ATP6AP1 was 0.792 (Fig. 7D). Further analysis implied that the level of ATP6AP1 is not correlated with CRC malignancy in lymph node invasion status and tumor metastasis (Fig. 7E,F, Table 2). Analysis of 90 patients with CRC confirmed that the level of ATP6AP1 was strongly related with OS. Kaplan–Meier analysis of two independent cohorts indicated that CRC patients with higher amounts of ATP6AP1 presented shorter OS times (p = 0.031; HR = 2.99) (Fig. 7G), and the higher age showed worse prognosis (p = 0.037; HR = 2.88) (Fig. 7H). Our data revealed that ATP6AP1 might serve as a diagnostic and prognostic indicator among CRC patients.

**Table 2:** Clinicopathological characteristics of the CRC patients in tissue arrays.

Characteristics	Low-ATP6AP1	High-ATP6AP1	p-value	Statistic
n	39	51		
Sex, n (%)			0.650	0.206
Man	18 (20%)	26 (28.9%)		
Female	21 (23.3%)	25 (27.8%)		
Age, median (IQR)	69 (61, 79)	71 (60, 79.5)	0.839	
T, n (%)			0.463	1.544
T3	33 (36.7%)	47 (52.2%)		
T1	4 (4.4%)	2 (2.2%)		
T2	2 (2.2%)	2 (2.2%)		
N, n (%)			0.666	0.814
N1	9 (10%)	14 (15.6%)		
N2	5 (5.6%)	9 (10%)		
N0	25 (27.8%)	28 (31.1%)		
M, n (%)			0.348	0.88
M1	1 (1.1%)	5 (5.6%)		
M0	38 (42.2%)	46 (51.1%)		
Pathologic.stage, n (%)			0.812	0.056
Stage II	37 (41.1%)	50 (55.6%)		
Stage III	2 (2.2%)	1 (1.1%)		

## Discussion

Colorectal cancer ranks third in cancer incidence and fourth in cancer mortality^17^. Although clinical advances in treating colorectal cancer through medical techniques, surgical techniques, and chemotherapy^18^, there is currently no effective diagnostic method to monitor the progression or recurrence of CRC. Recent studies showed that ATP6AP1 plays a crucial role in some cancers, and its mutation can contribute to malignant growth and cancer development. ATP6AP1 was upregulated in breast cancer tissues, and higher ATP6AP1 expression was associated with poorer outcomes^15^. Besides, ATP6AP1 levels exhibited significant negative correlations with B cells, CD8 + T cells, macrophages, and Treg cells^16^. However, no studies have focused on the characteristics of ATP6AP1 in CRC. In this study, we described the expression of ATP6AP1 and its significant role in the growth and progression of CRC and established a genetic risk-scoring model.

In our study, we investigated the role of ATP6AP1 in colorectal cancer and revealed its prognostic value, functional enrichment pathways, methylation, tumor immune infiltration, and variance analysis between high and low ATP6AP1 expression groups by bioinformatic analysis. ATP6AP1 expression was remarkably increased in tumor tissues compared with normal tissues of CRC. ATP6AP1 expression was associated with M stages in COAD patients. Higher expression of ATP6AP1 was significantly correlated to worse OS, DSS, and PFI. Besides, high ATP6AP1 expression might associate with a higher clinical stage in CRC patients. Cells with abnormal methylation of specific genes are at high risk of cancer, and aberrant methylation of specific genes occurs during cell oncogenesis^19,20^. Methylation levels of four CpG islands, including cg00645049, cg17800714, cg18742441, and cg19168249, were associated with the prognosis (p-value < 0.05) in COAD. CRC tissue microassays confirmed that ATP6AP1 was highly expressed in tumor tissues, and high-level ATP6AP1 predicted a poor prognosis. Our results showed that patients with high ATP6AP1 expression in CRC had shorter survival times and were more likely to progress, and ATP6AP1 could be used as an independent risk factor for survival.

There is growing evidence indicating the crucial role of the immune microenvironment in the development of tumors^21^. The growth of cells is closely tied to tumor immunity, and any disruption in the immune microenvironment can expedite the advancement of cancer^22^. To identify the dynamic immune elements within the tumor microenvironment (TME) in patients with tumors, computer algorithms are now extensively employed for the analysis of transcriptomic data. In our study, we utilized the ssGSEA method to determine the percentage of immune cell infiltration and the ratio of immune/mesenchymal components in colorectal cancer (CRC) samples sourced from the TCGA database. By validating the prognostic biomarker known as ATP6AP1, we established its association with the immune regulation of TME in CRC patients.

Different mutational forms of ATP6AP1 were associated with the immune infiltration of immune cells in COAD and READ. Utilizing the TIMER2 tool, we confirmed that the immune infiltration levels of B cells, CD8 + T cells, CD4 + T cells, macrophage cells, neutrophil cells, and dendritic cells were associated with ATP6AP1 expression in READ and COAD. Next, we performed the correlations between chemokines and ATP6AP1 in CRC. We analyzed the correlations between ATP6AP1 expression and immune checkpoint molecules using spearman’s method. The results showed that ATP6AP1 expression levels were significantly related to most checkpoint molecules, including TNFRSF8, CD40, PDCD1, TNFRSF18, TNFRSF25, TNFRSF4, TNFRSF14, VSIR, CD276, VTCN1, and BTLA in CRC. Furthermore, we analyzed correlations between ATP6AP1 and different chemokines in COAD and READ. The results showed significant (p-value < 0.05) and strong positive correlations in the following chemokines: CCL21, CXCL16, CXCL14, CX3CL1, CCL18, and CCL22. Besides, strong negative correlations with chemokines, including CXCL1-2/8, CXCL11, CXCL17, CXCL13, and CXCL5 in COAD.

Cancer-associated fibroblasts (CAFs) are one of the most critical components in the tumor microenvironment and play an essential role in the occurrence and development of tumors^23,24^. CAFs do not exist as individual cells around tumors but interact with them to promote tumor growth and survival and maintain their malignant tendency^25^. CAFs communicate with other stromal cells and tumor cells by secreting various cytokines, inhibiting immune cell function, and promoting tumor development^26^. CAFs secrete a range of cytokines, such as TGF-β, inhibit DC maturation, and promote Treg differentiation^27–29^. Therefore, we analyzed the correlation between ATP6AP1 and CAFs through R and TIMER based on TCGA datasets and found a significant positive correlation between them in COAD and READ. The high ATP6AP1 expression was positively related to the markers of CAFs, such as ACAT2, S1004A, VIM, and PDGFRA/B in COAD and READ. Therefore, these results further confirmed that the ATP6AP1 was significantly correlated with immune infiltrating cells and CAFs in READ and COAD, indicating that ATP6AP1 may alter the cellular composition in the CRC tumor microenvironment, fostering an immunosuppressive milieu that promotes tumor progression.

However, our study has certain limitations. Primarily, the analysis was based mainly on the TCGA dataset, complemented by validation with only 90 pairs of clinical samples. Future studies with larger sample sizes are needed to corroborate our findings. Furthermore, the role of ATP6AP1 in regulating immune cell infiltration requires in-depth experimental validation. Lastly, we plan to investigate whether ATP6AP1 facilitates CRC proliferation through in vivo and in vitro experiments and to explore the underlying mechanisms. These areas will be the focus of our future research efforts.

In conclusion, our study revealed that overexpression of ATP6AP1 predicted poor prognosis and advanced clinical stage in CRC patients. ATP6AP1 was associated with tumor immune cell infiltration and immune checkpoint expression in CRC. Therefore, ATP6AP1 may play a pivotal role in modulating tumor immunity and proliferation, underscoring its potential as a valuable biomarker and therapeutic target in colorectal cancer (CRC).

## Material and methods

### Transcriptome data sources

We utilized the TCGA database (http://cancergenome.nih.gov/abouttcga) to download mRNA expression data of ATP6AP1 in COAD and READ. For comparability purposes, the RNA-seq data expression levels were normalized to transcripts per million (TPM). To facilitate analysis, the transcriptome data underwent transformation into Log2 form. Subsequently, the Wilcoxon rank-sum test was performed on different tumor types. A p-value threshold of less than 0.05 indicated a significantly differential expression between tumor and normal tissues. All data analysis procedures were executed employing R software (Version 4.1.1). For data visualization, box plots were generated using the R package "ggplot2".

### ROC and AUC

ROC analysis was conducted to assess the predictive value of ATP6AP1 for COAD and READ, using gene expression data from the TCGA database. The area under the curve (AUC) and ROC were employed for this purpose.

### Survival analysis

To calculate the hazard ratio (HR) and 95% confidence intervals, we performed univariate survival analysis. Furthermore, the survival analysis for overall survival (OS), disease-specific survival (DSS), and progression-free interval (PFI) of ATP6AP1 in COAD and READ was carried out using R packages such as "survminer" and "survival". This allowed us to compare the differences in survival. To assess the discrimination of the nomograms, we calculated the concordance index (C-index).

### Immune cells infiltration

Using ssGSEA, we analyzed the association between ATP6AP1 and 24 different types of immune cell infiltration in the COAD and READ immune microenvironment. Moreover, we also investigated the differences in immune cell abundance between the high and low ATP6AP1 expression groups using ssGSEA. To explore the correlation between ATP6AP1 expression and the infiltration of various immune cell types and cancer-associated fibroblasts (CAFs), we utilized TIMER2. Additionally, we investigated the correlation between somatic copy number alteration (SCNA) of ATP6AP1 and the immune abundance of 6 leukocytes using the 'SCNA module'.

### Analysis of mutation and methylation

The genetic alteration analysis of ATP6AP1 was conducted using the cBioPortal website (https://www.cbioportal.org/). This website offers visualization, analysis, and the option to download large-scale cancer genomics data sets. The analysis focused on the alteration frequency, mutation type, and copy number alteration (CNA) in both COAD and READ. To investigate the DNA methylation status of CpG sites in ATP6AP1, the MethSurv database (https://biit.cs.ut.ee/methsurv/) was utilized. Specifically, the ATP6AP1 CpG methylation status was analyzed in the COAD and READ TCGA datasets. Additionally, the prognostic value of the CpG methylation status of ATP6AP1 was assessed in samples from COAD and READ. Lastly, the impact of ATP6AP1 CpG methylation status on the overall survival (OS) of CRC patients was also evaluated.

### Protein–protein interaction (PPI) networks and functional enrichment analysis

We utilized the online database STRING (https://string-db.org) to construct the PPI network using DEGs that differentiate between high and low ATP6AP1 expression levels. This network was then visualized using the R packages "igraph" and "ggraph." The defined cut-off value was an interaction score of 0.4, based on the median confidence. For the analysis of DEGs between the high- and low-ATP6AP1 groups, we employed the cluster Profiler R package. This package facilitated the investigation of Gene Ontology (GO) and Kyoto Encyclopedia of Genes and Genomes (KEGG) pathway enrichment.

### Tissue arrays and immunohistochemical (IHC) staining

The tissue microarrays of human CRC (ZL-Cocsur1801, N = 90) were obtained from Shanghai Zhuolibiotech Company Co., Ltd. (Shanghai, China). The array manufacturer provided the clinical and pathological information pertaining to the samples. The microarray underwent dewaxing in xylene baths for a total of three cycles, followed by rehydration using a graded series of alcohols. Subsequently, it was subjected to heat retrieval in a pressure cooker with sodium citrate buffer (pH 6.0) for 15 min. For IHC, the ATP6AP1 antibody (Proteintech, 15365-1-AP) was employed following the specified protocols. The staining scores for IHC (IS) were categorized into four ranks: 0 for negative, 1 for weak, 2 for moderate, and 3 for strong. To assess the percentage of positively stained cells (PS), we assigned scores as follows: 0 (< 5%), 1 (5–25%), 2 (25–50%), 3 (50–75%), and 4 (75–100%). The overall score for each slide was determined as the product of IS and PS, ranging from 0 to 12.

### Statistical analysis

Normalization of the data obtained from TCGA was carried out through log2 transformation. To assess the correlation between the two variables, we employed either Spearman's or Pearson's test. Various genetic correlation analyses were performed using R (version 3.6.3), along with the ggplot2 package. The TCGA dataset for COAD and READ was accessed through the following link: https://portal.gdc.cancer.gov/. For survival analysis, HRs and p-values were computed using either univariate Cox regression analysis or Log-rank test. A significance threshold of p-value < 0.05 was adopted for all statistical analyses.

### Statement

Our research involves human tissues derived from tumor and adjacent normal tissues of CRC patients. All patients signed an informed consent form. Ethical approval for this study was obtained from the Ethical Committee of Medical Research, Jiangsu Province Hospital of Nanjing Medical University (2018-SR-258). There are no clinical trials or animal experiments in our research. All experiments were performed in accordance with relevant guidelines and regulations.

### Informed consent

Written informed consent for publication was obtained from all participants.

### Supplementary Information

Supplementary Information.

## Data Availability

The datasets generated and analyzed during this study are available in the public database TCGA. The data that support the findings of this study are available from the corresponding author upon reasonable request.
