# Supplementary material for: ATP6AP1 as a potential prognostic biomarker in CRC by comprehensive analysis and verification
Source: Sci Rep. 2024 Feb 18;14:4018. doi: 10.1038/s41598-024-54437-7 (PMC10874971; doi:10.1038/s41598-024-54437-7)
Supplement: Supplementary file 1 — Supplementary Information. [file 41598_2024_54437_MOESM1_ESM.docx]

**Supplementary material**

**ATP6AP1 as a Prognostic Biomarker Correlated with Immune Infiltrates in colorectal Cancer**

**Supplementary figures**

**Figure S1. Construction of the nomogram to predict overall survival of COAD and READ patients based on TCGA dataset.** (A) The nomogram for predicting survival rates of READ patients in 1-, 3-, and 5 years. (B) The calibration curves showed COAD patient survival at 1-, 3- and 5 years.


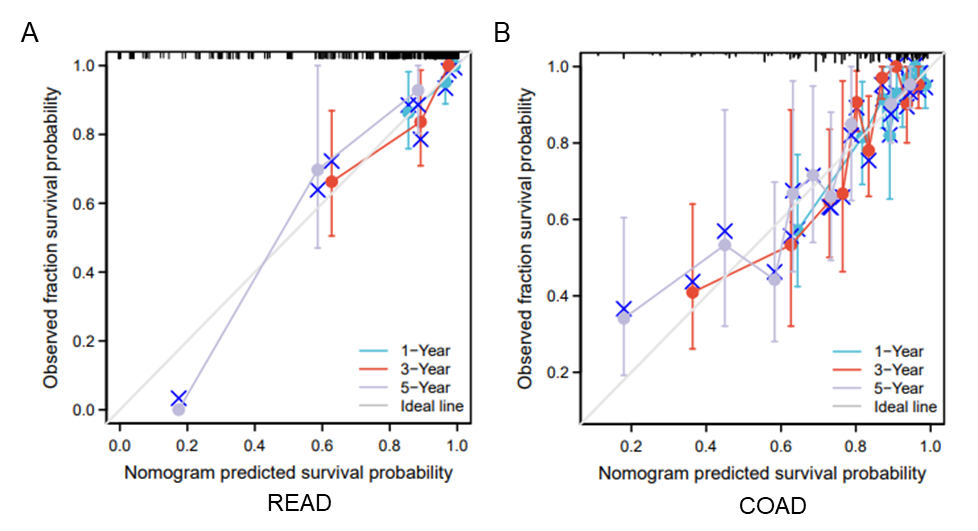


**Figure S2. The heatmaps show the expression of specific mRNAs in the READ (A) and COAD (B) patients with high- and low-ATP6AP1 expression from the TCGA dataset.**


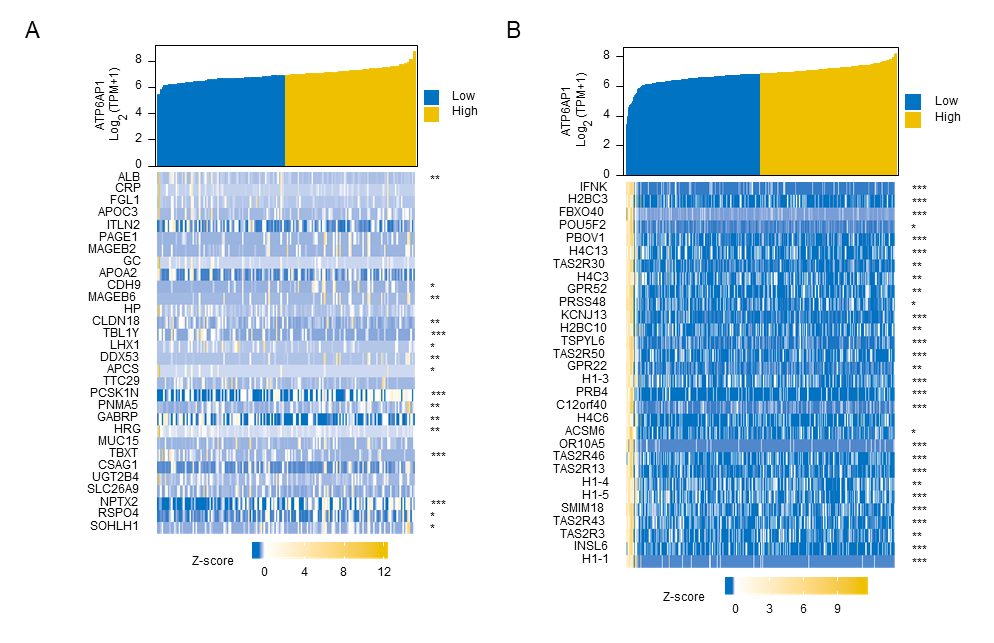


**Figure S3. The association of ATP6AP1 expression between relapse (R), stage (T), and gender.** (A) IHC scores of ATP6AP1 in CRC relapse (R1) and non-relapse (R0). (B) IHC scores of ATP6AP1 in CRC TI-II and TIII-IV. (C) The relationship between OS and gender (p-value = 0.894).


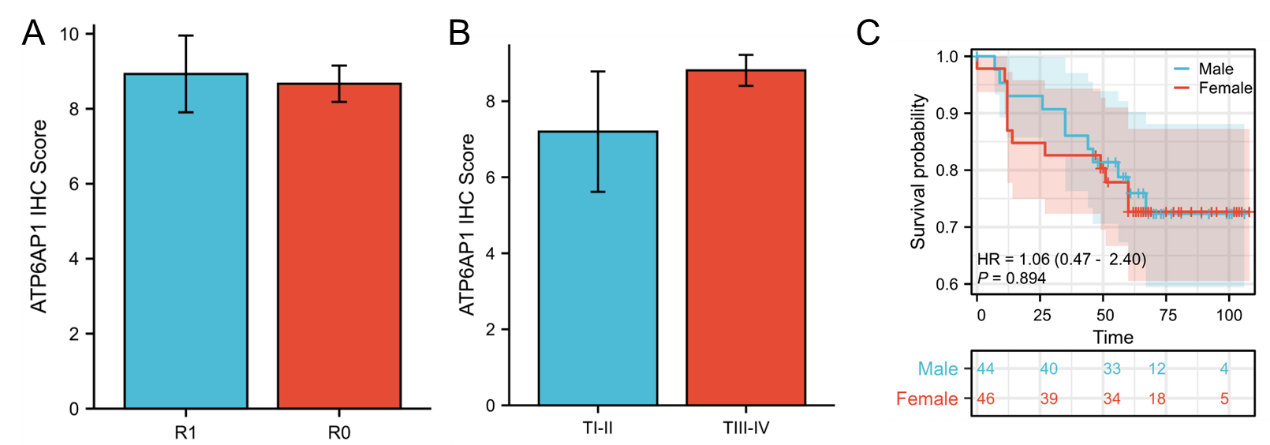


Table S1. Paired difference analysis of ATP6AP1 expression in pan-cancer.

|  |  |  |  |  |  |  |
| --- | --- | --- | --- | --- | --- | --- |
| Tumor | group | n |  | Tumor | group | n |
| BLCA | Normal | 19 |  | LUSC | Normal | 49 |
| BLCA | Tumor | 19 |  | LUSC | Tumor | 49 |
| BRCA | Normal | 113 |  | PAAD | Normal | 4 |
| BRCA | Tumor | 113 |  | PAAD | Tumor | 4 |
| CESC | Normal | 3 |  | PCPG | Normal | 3 |
| CESC | Tumor | 3 |  | PCPG | Tumor | 3 |
| CHOL | Normal | 8 |  | PRAD | Normal | 52 |
| CHOL | Tumor | 8 |  | PRAD | Tumor | 52 |
| COAD | Normal | 41 |  | READ | Normal | 9 |
| COAD | Tumor | 41 |  | READ | Tumor | 9 |
| ESCA | Normal | 8 |  | SARC | Normal | 2 |
| ESCA | Tumor | 8 |  | SARC | Tumor | 2 |
| HNSC | Normal | 43 |  | SKCM | Normal | 1 |
| HNSC | Tumor | 43 |  | SKCM | Tumor | 1 |
| KICH | Normal | 24 |  | STAD | Normal | 27 |
| KICH | Tumor | 24 |  | STAD | Tumor | 27 |
| KIRC | Normal | 72 |  | THCA | Normal | 59 |
| KIRC | Tumor | 72 |  | THCA | Tumor | 59 |
| KIRP | Normal | 32 |  | THYM | Normal | 2 |
| KIRP | Tumor | 32 |  | THYM | Tumor | 2 |
| LIHC | Normal | 50 |  | UCEC | Normal | 23 |
| LIHC | Tumor | 50 |  | UCEC | Tumor | 23 |
| LUAD | Normal | 58 |  |  |  |  |
| LUAD | Tumor | 58 |  |  |  |  |
|  |  |  |  |  |  |  |

Table S2. Differential analysis of ATP6AP1 expression in pan-cancer.

|  |  |  |  |  |  |  |
| --- | --- | --- | --- | --- | --- | --- |
| Tumors | group | n |  | Tumors | group | n |
| ACC | Tumor | 79 |  | LUAD | Normal | 59 |
| BLCA | Normal | 19 |  | LUAD | Tumor | 539 |
| BLCA | Tumor | 412 |  | LUSC | Normal | 49 |
| BRCA | Normal | 113 |  | LUSC | Tumor | 502 |
| BRCA | Tumor | 1113 |  | MESO | Tumor | 87 |
| CESC | Normal | 3 |  | OV | Tumor | 381 |
| CESC | Tumor | 306 |  | PAAD | Normal | 4 |
| CHOL | Normal | 9 |  | PAAD | Tumor | 179 |
| CHOL | Tumor | 35 |  | PCPG | Normal | 3 |
| COAD | Normal | 41 |  | PCPG | Tumor | 184 |
| COAD | Tumor | 480 |  | PRAD | Normal | 52 |
| DLBC | Tumor | 48 |  | PRAD | Tumor | 501 |
| ESCA | Normal | 11 |  | READ | Normal | 10 |
| ESCA | Tumor | 163 |  | READ | Tumor | 167 |
| GBM | Normal | 5 |  | SARC | Normal | 2 |
| GBM | Tumor | 169 |  | SARC | Tumor | 263 |
| HNSC | Normal | 44 |  | SKCM | Normal | 1 |
| HNSC | Tumor | 504 |  | SKCM | Tumor | 472 |
| KICH | Normal | 25 |  | STAD | Normal | 32 |
| KICH | Tumor | 65 |  | STAD | Tumor | 375 |
| KIRC | Normal | 72 |  | TGCT | Tumor | 156 |
| KIRC | Tumor | 541 |  | THCA | Normal | 59 |
| KIRP | Normal | 32 |  | THCA | Tumor | 512 |
| KIRP | Tumor | 291 |  | THYM | Normal | 2 |
| LAML | Tumor | 150 |  | THYM | Tumor | 120 |
| LGG | Tumor | 532 |  | UCEC | Normal | 35 |
| LIHC | Normal | 50 |  | UCEC | Tumor | 554 |
| LIHC | Tumor | 374 |  | UCS | Tumor | 57 |
|  |  |  |  | UVM | Tumor | 80 |
|  |  |  |  |  |  |  |
